# Direct Transmission of Severe Fever with Thrombocytopenia Syndrome Virus from Domestic Cat to Veterinary Personnel

**DOI:** 10.3201/eid2612.191513

**Published:** 2020-12

**Authors:** Atsushi Yamanaka, Yumi Kirino, Sho Fujimoto, Naoyasu Ueda, Daisuke Himeji, Miho Miura, Putu E. Sudaryatma, Yukiko Sato, Hidenori Tanaka, Hirohisa Mekata, Tamaki Okabayashi

**Affiliations:** Miyazaki Prefectural Miyazaki Hospital, Miyazaki, Japan (A. Yamanaka, S. Fujimoto, N. Ueda, D. Himeji);; University of Miyazaki, Miyazaki (Y. Kirino, P.E. Sudaryatma, Y. Sato, H. Tanaka, H. Mekata, T. Okabayashi);; Miyazaki Prefectural Institute for Public Health and Environment, Miyazaki (M. Miura)

**Keywords:** severe fever with thrombocytopenia syndrome, SFTS, severe fever with thrombocytopenia syndrome virus, SFTSV, viruses, Dabie bandavirus, cat, cat-to-human transmission, nosocomial outbreak, animal hospital, veterinary medicine, zoonoses, Japan

## Abstract

Two veterinary personnel in Japan were infected with severe fever with thrombocytopenia syndrome virus (SFTSV) while handling a sick cat. Whole-genome sequences of SFTSV isolated from the personnel and the cat were 100% identical. These results identified a nosocomial outbreak of SFTSV infection in an animal hospital without a tick as a vector.

Severe fever with thrombocytopenia syndrome (SFTS) is caused by the species *Dabie bandavirus* (family *Phenuiviridae*, genus *Bandavirus*), generally called severe fever with thrombocytopenia syndrome virus (SFTSV) (*1**,**2*). Cases of SFTS were identified in patients in China during 2009 (*3*) and subsequently in Japan and South Korea (*2*,*4*). Clinical signs include high fever, fatigue, gastrointestinal symptoms, neurologic symptoms, thrombocytopenia, leukocytopenia, and multiorgan failure (*5*). SFTS is potentially fatal, and mortality rates have reached 27% in Japan (*6*). Although the clinical information regarding SFTS in most animals is unclear, cats show fatal symptoms similar to those in humans (*7*). Enzootic SFTSV transmission is primarily tickborne; tick bites can also spread the virus to humans (*8*) and animals (*9*). Human-to-human transmission occurs rarely through contact with infected blood, body fluids, or mucus (*10*) and possibly by aerosols (*11*). In this study, we provide evidence for the direct cat-to-human transmission of the virus, leading to a nosocomial outbreak of SFTSV infection.

## The Study

Confirmatory testing of veterinary personnel samples was performed at the Laboratory of Microbiology, Miyazaki Prefecture Institute for the Public Health and Environment, Miyazaki, Japan. Cat sample analysis was performed at the Center for Animal Disease Control, University of Miyazaki. A 1-year-old male domestic cat was hospitalized on August 15, 2018, with jaundice, poor appetite, vomiting, and a rectal temperature of 40.4°C. Hematologic examination showed leukocytopenia (1,080 cells/μL, reference range 4–30 × 10^3^ cells/μL), thrombocytopenia (19,000 cells/μL, reference range 9–90 × 10^4^ cells/μL), and an increased level of total bilirubin (3.1 mg/dL, reference range 0–0.5 mg/dL) (*12*) (Table). The cat died 3 days after hospitalization.

**Table:** Hematologic and diagnostic results from a nosocomial outbreak of infection with severe fever with thrombocytopenia syndrome virus in animal hospital, Japan, 2018*

Characteristic	Cat,† Aug 15	Patient 1							Patient 2	
		Aug 27	Aug 28	Aug 29	Aug 30	Sep 5	Sep 11		Aug 28	Sep 11	
RT-PCR	+	−	−	+	+	ND	ND		+	ND	
Virus-specific IgG	+	−	−	ND	−	ND	+		−	+	
Real-time RT-PCR, copies/mL	1.5 × 10^11^	ND	ND	3.9 × 10^6^	6.0 × 10^6^	ND	ND		5.7 × 10^6^	ND	
Isolation‡	J1	ND	ND	J1	J1	ND	ND		J1	ND	
Leukocytes/μL	1, 080 (4–30 x 10^3^)	1,970	1,300	1,060	1,450	2,570	4,070		2,850	4,630	
Hemoglobin, g/dL	14.6 (9–18)	13.1	12.6	12.3	13.4	11.6	12.6		13.4	13.1	
Platelet count/μL	19,000 (9–90 x 10^4^)	81,000	63,000	53,000	59,000	155,000	214,000		254,000	261,000	
Total bilirubin, mg/dL	3.1 (0–0.5)	0.36	0.26	ND	0.28	0.44	0.69		0.44	0.42	
AST, IU/L	ND	18	17	20	27	51	11		25	24	
ALT, IU/L	91 (47.4–97.3)	12	10	12	16	60	25		37	28	
LDH, IU/L	ND	134	123	149	157	130	156		213	267	
C-reactive protein, mg/dL	ND	0.04	0.04	ND	0.03	0.01	0.002		0.17	0.19	

*ALT, alanine aminotransferase; AST, aspartate aminotransferase; J1, J1 genotype; LDH, lactate dehydrogenase; ND, not done; RT-PCR, reverse transcription PCR; –, negative; +, positive. †Values in parentheses are standard feline hematologic parameters reported by O’Brien et al. (*12*). ‡Virus isolated on Vero cells and genotyping.

Serum samples, saliva samples, and anal swab specimens (sampled on the first day of hospitalization) were sent to the Center for Animal Disease Control, University of Miyazaki, for molecular test targeting the small segment RNA of SFTSV by reverse transcription PCR (RT-PCR) and real-time RT-PCR (*3*). The amounts of SFTSV RNA were quantified as RNA copies per milliliter of serum. We detected a viral load of 1.5 × 10^11^ copies/mL (Table).

During hospitalization, the cat came into contact with a veterinarian (44-year-old woman) and a veterinary technician (20-year-old woman). During contact, both veterinary personnel wore protective clothing (gloves and surgical masks), but their eyes remained unprotected; they were not bitten or scratched by the cat. In addition, neither was bitten by ticks.

**Figure 1:**
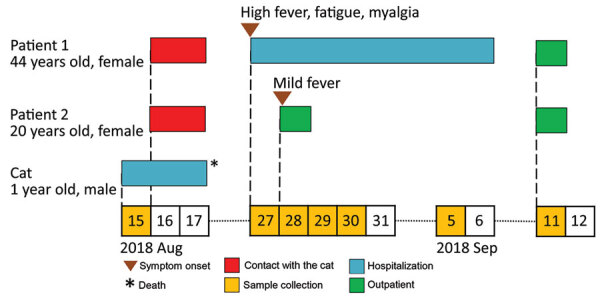
Timeline for transmission of severe fever with thrombocytopenia syndrome virus from cat to veterinary personnel in animal hospital, Japan, 2018. Patient 1, veterinarian; patient 2, veterinary technician.

After the death of the cat, symptoms consistent with SFTS developed in both veterinary personnel (Figure 1). Ten days after the death of the cat, on August 27, the veterinarian (patient 1) was hospitalized with a high fever (body temperature 39.2°C), fatigue, widespread myalgia, ocular pain, and bicytopenia. No abnormal symptoms were noted on cardiac, pulmonary, or abdominal examination. Hematologic examinations showed leukocytopenia and thrombocytopenia. On postadmission days 2 and 3, the presence of SFTSV RNA was confirmed in the serum samples by RT-PCR and real-time PCR (day 2, 3.9 × 10^6^ virus RNA copies/mL; day 3, 6.0 × 10^6^ virus RNA copies/mL) (Table). By postadmission day 10, the symptoms of SFTS abated, and patient 1 was discharged. Five days after discharge (September 11, 2018), SFTSV-specific IgG were detected in serum samples (*13*) (Table).

Eleven days after the death of the cat, on August 28, the veterinary technician (patient 2) also had fever and general malaise but less severe leukocytopenia. Serum samples collected from patient 2 were positive for SFTSV RNA by RT-PCR, and SFTSV RNA copies were quantified by using real-time RT-PCR (5.7 × 10^6^ virus RNA copies/mL) (Table). However, patient 2 recovered without being hospitalized. Similar to patient 1, IgG against SFTSV was present in serum collected from patient 2 on September 11.

We also isolated the virus. Vero cells were inoculated with SFTSV-positive serum samples taken from the cat, patient 1, and patient 2. The cells were adjusted to 10^5^ cells/mL and seeded onto a 12-well plate (Sumilon, http://www.sumilon.com) overnight as a monolayer (>60% confluence). A total of 200 μL of serum samples was inoculated into the cells. For all 3 serum samples (cat, patient 1, and patient 2), extensive cytopathic effects were observed after 3 days of incubation, and a high copy number of SFTSV RNA was detected in the cell supernatants.

**Figure 2:**
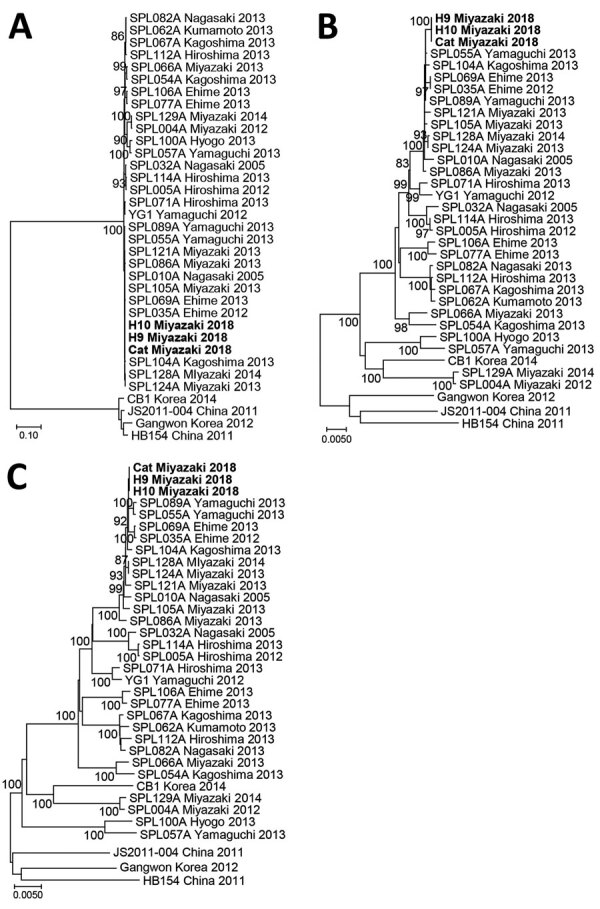
Phylogenetic analyses of severe fever with thrombocytopenia syndrome virus strains obtained from a cat and veterinary personnel in animal hospital, Japan, 2018. A) Small; B) medium; and C) large viral genomic RNA segments. Bold indicates H9/Miyazaki/2018 (from patient 1), H10/Miyazaki/2018 (from patient 2), and cat/Miyazaki/2018 (from cat). Scale bars indicate nucleotide substitutions per site.

Whole-genome sequencing (MiSeq; Illumina, https://www.illumina.com) of the viruses (named Cat/Miyazaki/2018, H9/Miyazaki/2018, and H10/Miyazaki/2018) was conducted as described (*14*), and sequences were submitted to DDBJ (accession nos. LC462229–37). For each viral RNA segment (small, medium, and large), the viral sequences from the cat and the 2 veterinary personnel showed 100% homology (Figure 2) and were closely related to the reference SFTSV strain YG1 from Japan (YG1/Yamaguchi/2012, accession nos. AB817995, AB817997, and AB817999). Furthermore, the sequence of the small segment was closely related to the SFTSV strains SPL128A Miyazaki 2014 and SPL124A Miyazaki 2013 (Figure 2, panel A), which were obtained from SFTS patients in the same prefecture during 2013–2014. Sequences of the medium and large segments were more distantly related to the SPL128A Miyazaki 2014 and SPL124A Miyazaki 2013 viruses, suggesting that they might have evolved from these strains (Figure 2, panels B, C).

SFTS is an emerging epizootic infectious disease and is transmitted primarily by ticks. However, some cases of SFTS do not involve ticks, and human-to-human transmission by aerosols (*10*) or through contact with infected blood or other body fluids (*6*,*9*) has been reported. Furthermore, a transmission route of SFTSV from a cat to a human has been confirmed with a partial nucleotide sequence of SFTSV in serum samples (*15*). In this report, we demonstrated a direct cat-to-human nosocomial outbreak of SFTSV with the following evidence: SFTSV was isolated from serum samples obtained from a cat and 2 veterinary personnel; the complete nucleotide sequence (segments small, medium, and large) of SFTSV from the cat and the 2 veterinary personnel showed 100% identity; the veterinary personnel were not bitten by ticks, nor were they bitten or scratched by the cat; and SFTS-like symptoms developed in the 2 veterinary personnel »10 days after close contact with the cat.

## Conclusions

Our results show that SFTSV can be transmitted to humans in the absence of ticks and that wearing limited protective clothing (e.g., face masks and rubber gloves) is insufficient to protect veterinary personnel from infection when handling infected animals. It is likely that cat-to-human transmission occurred by aerosols or contact with infected cat blood or other body fluids. This study draws attention to occupational exposure to potentially fatal zoonotic pathogens and highlights the need for stringent biosafety measures (i.e., personal protective clothing and equipment) to be in place when handling animals with symptoms of SFTS. These measures should include protection against aerosols that can be generated during treatment.
